# Machine Learning-Driven Innovations in Microfluidics

**DOI:** 10.3390/bios14120613

**Published:** 2024-12-13

**Authors:** Jinseok Park, Yang Woo Kim, Hee-Jae Jeon

**Affiliations:** 1 Department of Smart Health Science and Technology, Kangwon National University, Chuncheon 24341, Republic of Korea; 2 Department of Mechanical and Biomedical Engineering, Kangwon National University, Chuncheon 24341, Republic of Korea

**Keywords:** microfluidic devices, machine learning, droplet generation, biosensing technology

## Abstract

Microfluidic devices have revolutionized biosensing by enabling precise manipulation of minute fluid volumes across diverse applications. This review investigates the incorporation of machine learning (ML) into the design, fabrication, and application of microfluidic biosensors, emphasizing how ML algorithms enhance performance by improving design accuracy, operational efficiency, and the management of complex diagnostic datasets. Integrating microfluidics with ML has fostered intelligent systems capable of automating experimental workflows, enabling real-time data analysis, and supporting informed decision-making. Recent advances in health diagnostics, environmental monitoring, and synthetic biology driven by ML are critically examined. This review highlights the transformative potential of ML-enhanced microfluidic systems, offering insights into the future trajectory of this rapidly evolving field.

## 1. Introduction

Microfluidics has become a pivotal technology in contemporary biomedical research, enabling precise control and manipulation of fluids at the micro-scale [1,2,3,4,5]. This capability has revolutionized various domains, including diagnostics, drug delivery, and cellular biology. Microfluidic applications have diversified since the inception of the laboratory-on-a-chip (LOC) concept in 1979, encompassing chemical synthesis, single-cell analysis, and point-of-care testing [6,7,8]. The development of the micro total analysis system (µTAS) in 1994 marked a significant milestone, catalyzing further advancements in this field [9,10]. Subsequent breakthroughs, such as introducing microfluidic cell culture platforms in 2004, have enabled researchers to simulate physiological conditions and investigate cellular dynamics within controlled environments [11,12,13]. The integration of three-dimensional (3D) printing with microfluidic fabrication in 2008 significantly enhanced design flexibility, facilitating rapid prototyping of complex systems [14,15]. These technological strides have extended the capabilities of microfluidic systems and unlocked new possibilities in personalized medicine and real-time diagnostics.

Machine learning (ML) has also undergone transformative developments, reshaping data processing and analysis across scientific disciplines. Early ML models, including the recurrent neural network (RNN) in 1986 and long short-term memory (LSTM) networks in 1997, introduced novel approaches for handling sequential data and processing complex information in microfluidic systems [16,17,18]. The emergence of deep learning frameworks, such as Deep Belief Networks (DBNs) in 2006, AlexNet in 2012, and ResNet in 2015, has further expanded the capabilities of researchers to analyze high-dimensional datasets generated from microfluidic experiments [19,20,21]. These advancements in artificial intelligence (AI) have significantly enhanced the automation, speed, and precision of tasks such as image analysis, classification, and prediction, which are critical for interpreting complex experimental data [22,23,24].

The convergence of microfluidics and ML has led to the emergence of “intelligent microfluidics”, enabling the development of automated, highly efficient systems capable of independently analyzing, interpreting, and optimizing experimental processes [25,26]. These intelligent devices integrate ML algorithms to perform tasks that previously required manual supervision or expert input. For instance, droplet-based microfluidic systems now leverage intelligent algorithms to classify and sort droplets in real time using image data [26], while AI-powered biosensors enable rapid and precise biomarker detection, which is essential for early disease diagnosis and monitoring [25,26,27]. Furthermore, intelligent microfluidic systems facilitate real-time biological sample analysis, automate microfluidic channel design, and enhance droplet management for biochemical assays.

The application of intelligent microfluidics extends across diverse fields. AI-enhanced biosensors improve diagnostic accuracy by efficiently detecting and classifying biological samples [25,28]. In synthetic biology, these platforms automate complex synthesis processes, such as nanoparticle fabrication and cytokine storm monitoring in COVID-19 patients [29]. Additionally, intelligent systems drive advancements in skin-interfaced microfluidic devices for non-invasive health monitoring, which collect biomarker data from sweat and other body fluids [30,31]. Innovations in flow sculpting and droplet microfluidics further expand research opportunities, offering new insights into cellular dynamics and optimizing material synthesis, contributing to pharmaceutical and tissue engineering breakthroughs. A 2023 study exemplifies the potential of ML in optimizing channel design and device parameters, improving nanoparticle synthesis within microfluidic platforms [32,33].

**Figure 1:**
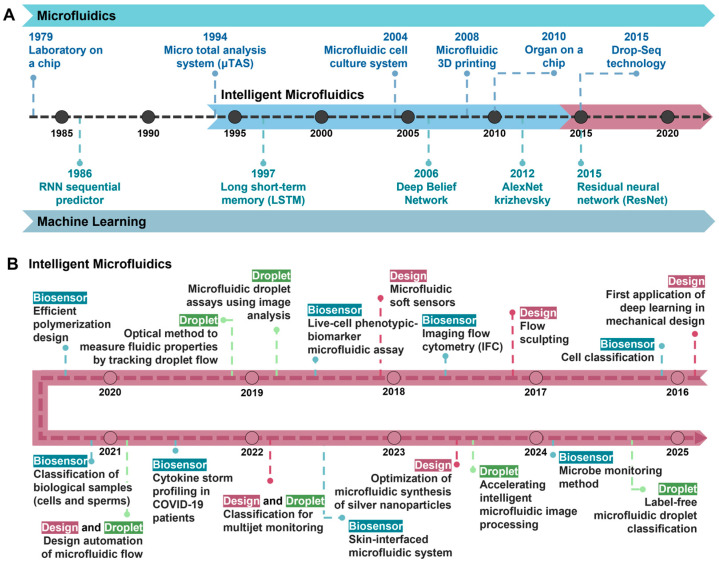
A timeline depicting the integration of microfluidics and machine learning biosensing applications. (**A**) The timeline of technological advancements in microfluidics and machine learning, highlighting key milestones and their integration. (**B**) Specific breakthroughs and applications in intelligent microfluidics.

Incorporating deep learning has transformed traditional engineering methods in microfluidic system design and functionality, as shown in Figure 1. For example, intelligent design automation optimizes system performance without requiring repetitive prototyping, reducing development time and costs. This advancement proves particularly advantageous for applications requiring custom microfluidic solutions, such as organ-on-chip systems and lab-on-a-chip diagnostics, where devices must be tailored to meet specific experimental needs. Intelligent microfluidic systems leverage ML for real-time decision-making and optimization, streamlining laboratory workflows, enhancing diagnostic accuracy [34,35], and advancing personalized medicine by customizing treatments for individual patients through real-time biomarker analysis.

This paper provides a comprehensive overview of both historical developments and recent innovations in microfluidics, highlighting the transformative impacts of intelligent technologies in this field. The evolution of microfluidic devices is examined, from simple fluid manipulation tools to advanced platforms capable of complex data analysis and decision-making, by reviewing key milestones. The integration of ML into microfluidics has not only propelled biomedical research but also laid the groundwork for a new era of autonomous, high-precision diagnostics and bioengineering solutions. This review offers valuable insights into the trajectory of microfluidics and intelligent microfluidics, guiding researchers and engineers in developing novel applications and next-generation technologies.

## 2. Advancing Microfluidic Device Design

The incorporation of ML in optimizing the design of microfluidic devices has opened new possibilities for improving the efficiency and performance of these systems [25]. ML’s predictive and analytical capabilities enable researchers to analyze complex datasets, predict outcomes, and fine-tune device parameters to meet specific performance objectives. ML proves particularly beneficial in predicting fluid flow dynamics, optimizing microchannel configurations, and refining the overall architecture of microfluidic devices [36,37]. Following a review of the fabrication methods and design considerations of existing microfluidic devices, this section will delve into research studies that have successfully integrated machine learning techniques to enhance device performance.

### 2.1. Techniques in Microfluidic Fabrication

Fabricating microfluidic devices requires techniques that ensure high precision and adaptability in constructing micro-scale structures for various applications. The most widely used methods include photolithography, soft lithography, and 3D printing, each chosen based on specific application needs and material properties [38,39,40].

Photolithography is favored for its ability to produce intricate, high-precision microstructures. This technique involves transferring a pattern from a photomask onto a substrate, typically silicon or glass coated with a photoresist through controlled light exposure [41,42,43]. Its accuracy makes it ideal for applications requiring complex channel designs and detailed layouts. However, photolithography is associated with high production costs and demands specialized equipment, limiting accessibility for resource-constrained laboratories and large-scale manufacturing. Soft lithography, especially polydimethylsiloxane (PDMS)-based molding, provides a more adaptable and cost-effective alternative [44,45]. A silicon master fabricated through photolithography serves as a mold in this process to create PDMS replicas. PDMS is highly suitable for biological applications due to its biocompatibility and optical transparency [45,46]. Moreover, soft lithography supports rapid prototyping, enabling researchers to swiftly iterate and refine designs, which is particularly advantageous in experimental settings that require continuous testing and development.

In addition to these methods, CNC machining, injection molding, and hot embossing offer further versatility in fabricating microfluidic devices. CNC machining is recognized for its high precision and ability to customize intricate designs, although it can be costly for large-scale production [47,48]. Injection molding provides a high-throughput solution ideal for commercial applications, as it allows for rapid, consistent replication once a mold is created, though initial mold fabrication is expensive. Hot embossing, which involves pressing a mold onto a thermoplastic substrate under heat and pressure, strikes a balance between scalability and precision, making it suitable for moderate-scale applications [47,49]. Including these fabrication methods widens the range of available options, offering tailored solutions for various cost, scalability, and precision requirements.

Recently, 3D printing, also known as additive manufacturing, has gained prominence due to its capacity to produce intricate 3D structures in a single fabrication step. Three-dimensional printing within microfluidics facilitates rapid fabrication of customized devices with geometries that are challenging to achieve using conventional methods. Techniques such as stereolithography (SLA) and digital light processing (DLP) are well suited for microfluidic applications, offering high resolutions and material versatility [50,51,52,53,54]. The primary advantages of 3D printing include design flexibility, reduced fabrication time, and the ability to incorporate intricate features, such as interconnected channels and multilateral designs, without additional assembly steps. Collectively, these fabrication techniques have advanced the development of microfluidic devices by providing solutions tailored to various requirements, including material compatibility, resolution, scalability, and cost [54,55,56]. Researchers and engineers have used these diverse approaches to broaden the scope of microfluidics, enabling its application across multiple domains, ranging from biomedical diagnostics to chemical analysis.

### 2.2. Design Considerations for Microfluidic Devices

Effective design of microfluidic devices demands careful attention to several critical factors to ensure optimal performance and adaptability for specific applications. Key design elements include fluid dynamics, device scalability, and the integration of electronic components, all of which play a pivotal role in determining the functionality and usability of a device. Fluid dynamics constitutes one of the most significant aspects of microfluidic design because fluid behavior at the micro-scale differs markedly from that at the macro-scale [57,58]. Laminar flow predominantly governs fluid movement in microfluidic devices with minimal turbulence. Understanding and controlling laminar flow are essential for ensuring efficient device performance [59]. Parameters such as the Reynolds number, surface tension, and capillary effects directly influence fluid transport, affecting processes like mixing, separation, and reactions within microchannels. Precise regulation of fluid dynamics is crucial for biosensing and chemical analysis applications, where predictable and reproducible fluid behavior enhances accuracy and reliability.

Scalability represents another crucial factor, especially for high-throughput or commercial applications. While many microfluidic devices are initially developed for laboratory-scale experiments, transitioning to large-scale production introduces challenges related to fabrication costs, consistency, and reliability. Design strategies that promote modularity and manufacturability streamline scalability, enabling the adaptation of devices for mass production without compromising performance [59,60]. This aspect is essential for advancing microfluidic technologies from experimental prototypes to commercially viable products, particularly in diagnostics and drug development [60,61]. Integrating electronics has also become increasingly significant because modern microfluidic devices frequently incorporate sensors, actuators, and other electronic components to facilitate real-time monitoring and data acquisition. This electronic integration supports advanced functionalities, including automated control, data transmission, and feedback mechanisms, which enhance the versatility and user-friendliness of these systems. However, incorporating electronic elements introduces challenges, such as ensuring material compatibility, reducing signal interference, and optimizing the physical layout to maintain the compact structure of the device [60,62,63]. For instance, including electronic components in biosensing applications significantly improves detection accuracy and responsiveness by delivering immediate, high-resolution readouts.

Addressing these critical design considerations ensures the creation of efficient, reliable, and adaptable microfluidic devices. Researchers can enhance the functionality and practicality of these systems by systematically tackling challenges, making them suitable for both experimental research and scalable commercial applications.

### 2.3. Machine Learning in Design Optimization

**Figure 2:**
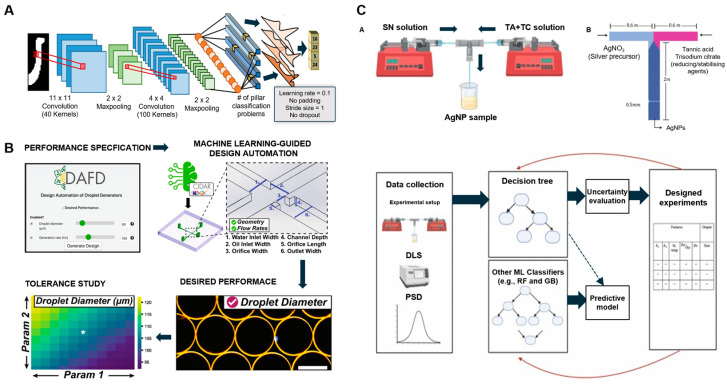
Leveraging machine learning for microfluidic device design optimization. (**A**) A schematic of the convolutional neural network (CNN) used in this work. Adapted with permission [65], copyright 2017, *Scientific Reports*. (**B**) The workflow of the developed design automation tool for flow-focusing droplet generators, called DAFD. Adapted with permission [64], copyright 2021, *Nature Communications*. (**C**) A workflow chart and a description of methods used in the building of the machine learning-guided design of an experiment based on the decision tree method. Adapted with permission [66], copyright 2023, *Chemical Engineering Research and Design*.

ML significantly enhances microfluidic design by enabling the prediction of fluid flow dynamics [25,64]. Models such as convolutional neural networks (CNNs) and RNNs have been employed to forecast fluid flow patterns within microfluidic channels [18]. These models analyze extensive datasets from simulations or experimental data to determine optimal channel geometries and flow rates tailored to specific applications. This predictive capability proves advantageous in biosensing, where precise fluid control directly influences the sensitivity and accuracy of the device, ensuring reliable diagnostic and analytical outcomes. For example, a 2017 study addressed the challenge of designing flow-sculpting devices to generate specific fluid flow patterns (Figure 2A). This study demonstrated how ML can circumvent traditional, labor-intensive design processes that demand substantial user input; efficiently approximate high-dimensional design spaces; and generate reliable out-of-sample predictions by leveraging deep learning to solve the inverse design problem [65].

ML also plays a pivotal role in optimizing droplet generation within microfluidic systems. A 2021 study introduced the DAFD tool, a web-based platform that automates droplet generator design by accurately predicting droplet size and the generation rate (Figure 2B). This tool minimizes the need for specialized expertise and iterative adjustments, facilitating the application of microfluidic platforms in life sciences, particularly in areas such as enzyme discovery and early cancer detection [64]. Additionally, ML has been employed to enhance silver nanoparticle (AgNP) synthesis within microfluidic systems, as demonstrated in a 2023 study (Figure 2C). This study used a T-junction microfluidic system integrated with ML to optimize AgNP synthesis by adjusting parameters such as the Reynolds number, Dean number ratio, and storage temperature, leading to improved particle stability. A decision-tree-guided design of experiments developed a predictive model for AgNP size, further refined through targeted experimental iterations [66]. ML models, including decision trees, random forests, and XGBoost, improve synthesis accuracy while reducing the need for extensive trial-and-error procedures.

Additionally, ML enhances the overall architecture of microfluidic devices by analyzing multiple design factors and predicting their interactions [25]. This comprehensive approach helps ML algorithms balance essential considerations, such as fluid dynamics and electronic integration, ensuring that sensors, actuators, and other components are optimally positioned within a device. Achieving this balance is crucial for advanced applications where complex interactions significantly impact device performance and accuracy. ML enables the development of sophisticated microfluidic devices that meet the rigorous demands of biosensing, diagnostics, and environmental monitoring by identifying ideal configurations [67,68]. Incorporating ML into design and optimization processes facilitates the production of highly efficient, application-specific microfluidic devices with enhanced precision, functionality, and adaptability [69]. As ML algorithms advance, their role in microfluidic design will become increasingly refined, paving the way for intelligent microfluidic systems capable of autonomous operation and real-time decision-making. This integration advances current microfluidic technologies and lays the groundwork for future innovations in autonomous and intelligent diagnostic systems.

### 2.4. Advanced Machine Learning Applications in Droplet-Based Microfluidics

The integration of ML and droplet-based microfluidics has transformed droplet control, classification, and automated design, significantly improving the precision, scalability, and adaptability of these systems [70,71]. Droplet-based microfluidics is widely used in diagnostics, drug discovery, and materials science, where precise droplet manipulation is essential for achieving consistent and reliable outcomes [27,72]. ML applications show that they address key challenges in droplet-based microfluidics, focusing on predictive modeling, real-time classification, and automated design, with relevant case studies highlighting these advancements.

**Figure 3:**
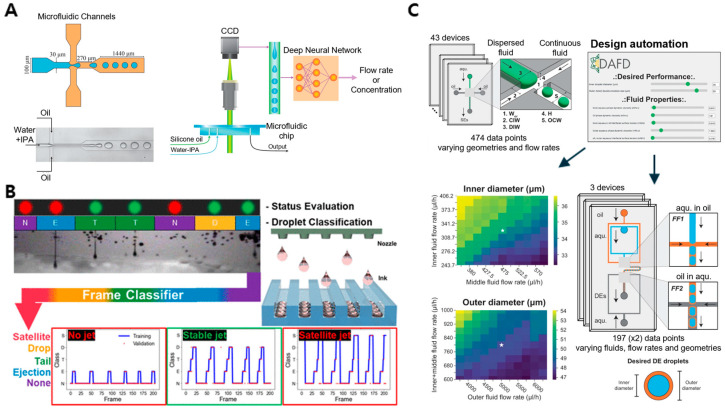
Machine learning-enhanced control and design of droplets in microfluidic systems. (**A**) Prediction of droplet flow dynamics using deep neural networks. Adapted with permission [65], copyright 2019, *Scientific Reports*. (**B**) Real-time droplet classification for multijet monitoring. Adapted with permission [64], copyright 2022, *ACS Applied Materials & Interfaces*. (**C**) Design automation of single- and double-emulsion droplets through machine learning. Adapted with permission [66], copyright 2024, *Nature Communications*.

A primary ML application in droplet-based microfluidics involves predicting droplet flow dynamics, a task complicated by the interplay of factors such as channel geometry, flow rates, and fluid properties. A 2019 study demonstrated the ability of deep neural networks to predict droplet flow patterns under varying conditions within microfluidic channels (Figure 3A) [73]. This predictive capability is particularly advantageous in drug delivery and chemical synthesis, where precise flow control is essential to ensure consistent droplet behavior and maintain reliable outcomes.

Real-time droplet classification plays a crucial role in multijet microfluidic systems, especially for high-throughput applications requiring continuous monitoring and precise control of droplet formation. A 2022 study developed a deep learning model tailored for droplet classification in multijet systems (Figure 3B), enabling the real-time assessment of droplet size, shape, and uniformity [74]. This ML-driven classification approach enhances quality control by ensuring consistent droplet characteristics, which are essential for applications such as diagnostic assays, cell analysis, and materials processing. Automating droplet classification improves reliability and enhances scalability, making droplet-based platforms more suitable for complex, high-throughput environments.

Moreover, ML has been instrumental in automating the design of single- and double-emulsion droplets, which is critical for scaling microfluidic applications across industries. Designing droplets with specific properties traditionally required iterative experimentation, which is labor-intensive and time-consuming. A 2024 study introduced an ML-based tool that automates emulsion droplet design (Figure 3C) and predicts essential parameters such as size and stability based on input conditions [75]. This tool significantly reduces trial-and-error experimentation, streamlining customized droplet production for industries including cosmetics, food science, and biomedical research [76]. ML-based automation enhances the efficiency and accessibility of droplet-based microfluidics for a range of industrial applications by simplifying droplet customization.

Incorporating various ML techniques, such as droplet flow prediction, real-time classification, and automated design, provides a comprehensive framework for droplet control in microfluidic platforms (Table 1) [27,64]. This integrated approach facilitates dynamic adjustments to droplet generation and manipulation based on real-time data and specific operational requirements. Achieving such precise control enables the development of autonomous droplet-based systems that can respond to changing conditions, improving both efficiency and precision. This holistic use of ML holds significant potential for further applications in diagnostics, high-throughput screening, and environmental monitoring, as it can ensure consistent, high-quality droplet production and control.

The evolving landscape of ML technologies suggests that their role in droplet-based microfluidics will continue to expand. Future studies should explore reinforcement learning and generative models to enhance systems’ adaptability and autonomy. These advanced ML models would enable continuous optimization and real-time decision-making capabilities, further refining droplet control and expanding the versatility of microfluidic platforms [77,78]. Additionally, integrating ML-enhanced microfluidic systems with the Internet of Things (IoT) and edge computing technologies could facilitate remote monitoring and control, aligning these platforms with the needs of complex applications such as environmental monitoring and point-of-care diagnostics [79].

**Table 1:** Overview of ML applications in microfluidic device design and droplet-based systems.

Application Field	Fabrication Technique	Machine Learning Method	Training Samples	Accuracy	Ref.
Design	-	CNN	150,000	94.52%	[80]
	Flow sculpting	CNN	250,000	-	[65]
	3D printing	RNN	2070	85.42%	[81]
	-	Design Automation of Fluid Dynamics (DAFD) Neural Optimizer	710	95.1%	[64]
	Inkjet printing	CNN	7852	90%	[74]
Droplet	-	Design Automation of Fluid Dynamics (DAFD) Neural Optimizer	710	95.1%	[64]
	-	-	7500	98%	[82]
	-	Deep neural network (DNN)	6000	97.1%	[73]
	Soft lithography	Supervised neural network	498,002	91.7%	[83]
	Computer numerical control (CNC) machining	Mask Region-based CNN (Mask R-CNN)	-	98%	[84]

## 3. Applications of Microfluidic Devices in Biosensing

ML significantly enhances the functionality of biosensors by improving data processing capabilities and enabling advanced predictive modeling [85]. Biosensors produce large volumes of high-dimensional data that are challenging to analyze with traditional methods, but ML algorithms, particularly those rooted in deep and statistical learning, extract meaningful patterns and features from these datasets. Techniques such as dimensionality reduction, feature selection, and data normalization are employed to eliminate noise, increasing the accuracy of biosensor readings [86,87]. These capabilities automate data analysis and facilitate real-time interpretation, which is critical in clinical and environmental monitoring contexts where accurate and timely data interpretation is essential for informed decision-making [87,88]. Furthermore, training ML models on extensive historical datasets enables researchers to develop algorithms capable of predicting outcomes based on new input variables [89]. For instance, regression techniques and classification algorithms, such as support vector machines and random forests, can predict the concentrations of specific analytes from biosensor signals, enhancing sensitivity and specificity while enabling dynamic adjustments to biosensor operations in response to evolving conditions. Through these integrated data processing and predictive modeling capabilities, ML strengthens the reliability and efficiency of biosensors across various applications, from healthcare diagnostics to food safety monitoring.

Microfluidic devices have revolutionized biosensing by enabling precise, real-time analysis with minimal sample volumes, making them suitable for health diagnostics, environmental monitoring, and synthetic biology (Table 2). The integration of ML enhances these systems, improving their data processing speed and accuracy. This section examines the application of microfluidic devices, explores recent technological advancements, and summarizes core techniques.

### 3.1. Health Monitoring and Diagnostics

**Figure 4:**
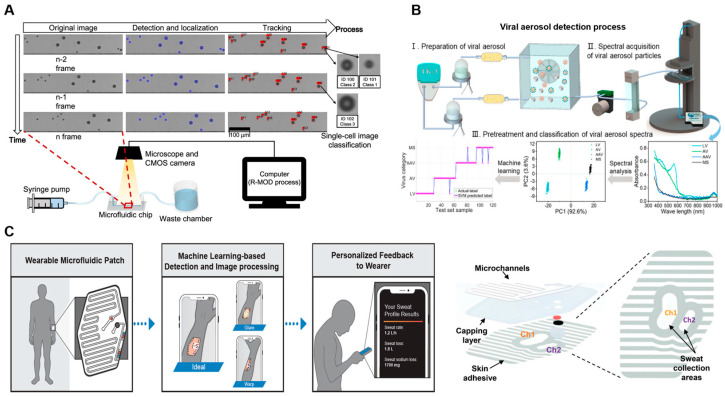
Machine learning-enabled real-time monitoring and analysis within microfluidic platforms. (**A**) Microscopy-based label-free imaging flow cytometry with real-time image processing. Adapted with permission [90], copyright 2017, *Scientific Reports*. (**B**) Viral aerosol detection employing microfluidics and machine learning for rapid classification. Adapted with permission [91], copyright 2024, *ACS Nano*. (**C**) Skin-interfaced microfluidic patch with machine learning-based image processing for sweat biomarker analysis. Adapted with permission [92], copyright 2022, *Advanced Materials Technologies*.

Integrating microfluidic systems with ML has advanced real-time monitoring of biochemical markers, contributing to personalized healthcare and fitness tracking. A 2022 study introduced a skin-interfaced microfluidic patch equipped with ML-based image processing for analyzing sweat biomarkers in real time [82]. This system effectively addresses challenges such as inconsistent lighting, variations in camera angles, and motion artifacts during image capture. ML algorithms filter out these noise factors, facilitating accurate measurement of biomarkers, including the chloride concentration, even in dynamic environments (Figure 4C). This system identifies patch boundaries, quantifies sweat volume, and analyzes chloride levels using computer vision, providing continuous insights into the hydration status and electrolyte balance of the wearer throughout physical activities. This wearable biosensing technology offers innovative solutions for continuous health monitoring, supporting applications such as metabolic tracking and personalized health optimization. Building on these advancements, this field continues to evolve, tackling global challenges.

Microfluidic devices are central to health diagnostics, enabling rapid and non-invasive analysis of biological samples. A study from 2017 introduced the “Real-time Moving Object Detector” (R-MOD) system for label-free imaging flow cytometry within a microfluidic chip (Figure 4A). This system allows cells to be analyzed in real time as they flow through a microfluidic channel, eliminating the need for dyes or labels. Using a multi-object tracking algorithm, the R-MOD system captures and counts individual cells under a microscope, while ML algorithms perform image analysis without labeling. Experimental results demonstrated a processing speed of 500 fps, with a mean average precision (mAP) of 93.3%, indicating both accuracy and stability in real-time cell monitoring [90]. This study also explored the use of a CNN (convolutional neural network) classifier to distinguish different types of white blood cells, including basophils, eosinophils, lymphocytes, monocytes, and neutrophils, based on low-resolution microscope images. The research results suggest that the CNN classifier can differentiate between cells with similar sizes but different morphologies. The classification accuracy can vary depending on factors such as the dataset, optimization methods, and neural network architecture. However, ongoing research in deep learning has provided a variety of classification algorithms that can accurately infer class categories from images, leading to improved accuracy.

Artificial intelligence (AI) is reshaping the medical field by automating complex tasks such as image segmentation and pattern recognition, thereby enhancing diagnostics, treatments, and overall patient care. A 2024 study conducted a comprehensive evaluation of AI models to address the classification of the presence of bubbles in microfluidic channels under diverse imaging conditions [93]. Among the machine learning (ML) models evaluated, the random forest algorithm achieved superior results, with 95.52% sensitivity, 82.57% specificity, and an AUC of 97%, surpassing other ML algorithms. For deep learning (DL) models, DenseNet169 emerged as the top performer, recording 92.63% sensitivity, 92.22% specificity, and an AUC of 92%. When integrated into a mobile POC system, DenseNet169 demonstrated excellent accuracy (>0.84), even under challenging imaging conditions, illustrating the transformative potential of AI in precision medicine and accessible diagnostics. This AI integration could lead to enhanced patient outcomes and streamlined healthcare services.

**Table 2:** Overview of ML techniques applied in microfluidic systems.

Application Field	Diagnostics	Fabrication Technique	Machine Learning Method	Ref.
Biosensor	Cell classification	-	Heuristic genetic algorithm	[94]
	Imaging flow cytometry (IFC)	-	Fully Convolutional Residual Networks (FCRNs) and CNN	[90]
	Live-cell phenotypic biomarker	-	-	[95]
	Obtain datasets on a complex chemical reaction	photopolymerization SLA	Artificial Neural Networks (ANNs)	[96]
	Classification of biological samples	SLA	-	[97]
	Lung cancer cells	-	CNN	[98]
	Cytokine storm profiling	Microfluidic patterning technique	CNN	[29]
	Sweat biomarkers	-	Machine learning-based and image analysis algorithms	[92]
	Airborne microbiological detection	Soft lithography	Principal Component Analysis (PCA)–support vector machine (SVM) model	[91]
	Complex reactive proteins (CRPs)	-	Particle Swarm Optimization (PSO)–Artificial Neural Network (ANN) model	[99]

### 3.2. Environmental Monitoring

Integrating microfluidic chips with machine learning has enabled efficient, real-time analysis and prediction, which supports continuous monitoring [100]. This convergence has spurred research focused on applications such as air quality detection, pollutant identification, and rapid hazard alert systems, particularly for monitoring harmful substances like CO_2_ and industrial gases. Microfluidic devices facilitate detection and analysis of airborne particles, pollutants, and various environmental contaminants [101]. A 2024 study introduced the minimum-minute ML microfluidic microbe monitoring method (M7) [91], which integrates a microfluidic chip with a spectrometer to capture aerosol spectral data in real time (Figure 4B). Designed to leverage inertial separation and laminar flow properties, the chip achieves a separation efficiency of 95.99% for particles approximately 2 μm in diameter. ML algorithms enable this system to classify aerosol particles within 30 min, significantly reducing processing time compared to traditional PCR methods. This system achieved a 97.87% accuracy rate for virus detection, providing a rapid and reliable solution for air quality assessment and pollutant monitoring. This approach is a valuable alternative to conventional laboratory-based techniques, streamlining environmental evaluations.

Atmospheric particulates, like pollen, impact air quality and health, making accurate detection crucial. A 2024 study introduced an automated system combining microfluidic technology and a smartphone-based photonic detection platform [102]. This low-cost solution (USD 50) integrates a compact optical microscopy module and a consumer-grade camera for community-driven environmental monitoring. This system allows real-time air microparticle sampling and detection via a microfluidic chip. The lightweight detection network “PollenDet” achieved a mean average precision (mAP) of 94.6%, offering valuable insights into airborne particulates and their health impacts. In addition, in 2024, microfluidic chips are crucial for studying CO_2_ displacement and gas–liquid exchange processes, offering insights for improving oil and gas recovery [103]. Traditional image processing struggles with complex gas–liquid dynamics, so this study proposed a deep learning-based bubble detection method for microfluidic experiments. By using CycleGAN to generate training samples and integrating improvements like the SE attention mechanism and Fast-PConv, this method achieved 94% detection accuracy with only 20 annotated images. DeepSORT tracks bubble movements, providing valuable insights for analyzing multiphase flow in porous media.

## 4. Future Aspects

The integration of ML into microfluidic systems is poised to drive transformative advancements across domains, such as healthcare, environmental monitoring, and synthetic biology [101,104]. The development of advanced ML algorithms, including reinforcement learning and generative models, is expected to further enhance the adaptability and autonomy of these systems. Such algorithms facilitate the real-time optimization of experimental conditions by enabling systems to learn from previous outcomes and autonomously implement intelligent adjustments without human intervention. The convergence of microfluidics with IoT technologies and edge computing will further elevate the utility of these platforms [105]. This combination enables remote monitoring and control, empowering microfluidic devices to perform real-time data analysis and informed decision-making. This capability enhances their applicability across diverse contexts, such as environmental monitoring and point-of-care diagnostics. For instance, portable microfluidic devices embedded with ML algorithms could deliver immediate results for air quality assessment or disease detection in remote locations, reducing dependence on conventional laboratory infrastructure [106,107,108].

Microfluidic systems integrated with ML hold significant potential for providing tailored healthcare solutions as personalized medicine gains momentum. These systems can facilitate continuous monitoring of biomarkers, enabling healthcare providers to personalize treatment plans based on real-time patient data. Such precision in therapeutic interventions enhances patient care, particularly for chronic disease management, where prompt modifications to treatment protocols can significantly improve health outcomes. However, despite these promising applications, several challenges remain for the real-world implementation of ML-integrated microfluidic devices. One major issue is scalability. Many microfluidic devices struggle to transition from lab-scale prototypes to large-scale production due to technical and cost-related constraints [109]. Addressing this challenge requires advancements in manufacturing techniques and the development of standardized protocols to ensure reproducibility and cost-efficiency on a commercial scale. Integration with existing healthcare systems presents another critical hurdle. For these devices to be effectively adopted, they must seamlessly interface with current infrastructure and workflows in hospitals and clinics. Designing adaptable plug-and-play interfaces capable of functioning within diverse medical environments could significantly facilitate this process [110]. In addition, concerns about data privacy and security must be addressed when handling sensitive patient information in ML-based systems. Robust data protection frameworks that comply with evolving regulations are essential to safeguard patient information and foster trust in these technologies [111]. Another pressing issue is the risk of model obsolescence in the face of rapid technological advancements in AI. Continuously updating ML models through modular system designs and cloud-based training infrastructures will be crucial to ensure that these systems remain effective and relevant over time [112].

Efforts to integrate multimodal data sources by combining biological, chemical, and physical parameters will establish more comprehensive analytical platforms. These advanced platforms will deepen our understanding of intricate biological systems, paving the way for breakthroughs in drug discovery, disease modeling, and synthetic biology [108,113]. The evolution of materials science will also play a crucial role because innovations in biocompatible materials and advanced manufacturing techniques will facilitate the development of microfluidic devices with superior functionalities. Overcoming these challenges through interdisciplinary research and strategic collaborations will ensure that intelligent microfluidic systems can achieve their full potential, providing transformative solutions across fields like healthcare and environmental science [114]. As these technological advancements converge, the potential for intelligent microfluidic systems to revolutionize fields such as biomedicine and environmental science continues to grow. This trajectory lays a strong foundation for innovative solutions and will significantly advance autonomous diagnostics and bioengineering, contributing to the future of precision science and healthcare.
